# The Common Cellular Events in the Neurodegenerative Diseases and the Associated Role of Endoplasmic Reticulum Stress

**DOI:** 10.3390/ijms23115894

**Published:** 2022-05-24

**Authors:** Soojeong Kim, Doo Kyung Kim, Seho Jeong, Jaemin Lee

**Affiliations:** 1 Department of New Biology, Daegu Gyeongbuk Institute of Science and Technology (DGIST), Daegu 42988, Korea; soojeong_kim@dgist.ac.kr (S.K.); dookyung@dgist.ac.kr (D.K.K.); jeongseho@dgist.ac.kr (S.J.); 2 New Biology Research Center, Daegu Gyeongbuk Institute of Science and Technology (DGIST), Daegu 42988, Korea; 3 Well Aging Research Center, Daegu Gyeongbuk Institute of Science and Technology (DGIST), Daegu 42988, Korea

**Keywords:** endoplasmic reticulum, ER stress, neurodegenerative disease, Alzheimer’s disease, Parkinson’s disease, Huntington’s disease, amyotrophic lateral sclerosis, prion disease, misfolded protein, unfolded protein response

## Abstract

Neurodegenerative diseases are inseparably linked with aging and increase as life expectancy extends. There are common dysfunctions in various cellular events shared among neurogenerative diseases, such as calcium dyshomeostasis, neuroinflammation, and age-associated decline in the autophagy-lysosome system. However, most of all, the prominent pathological feature of neurodegenerative diseases is the toxic buildup of misfolded protein aggregates and inclusion bodies accompanied by an impairment in proteostasis. Recent studies have suggested a close association between endoplasmic reticulum (ER) stress and neurodegenerative pathology in cellular and animal models as well as in human patients. The contribution of mutant or misfolded protein-triggered ER stress and its associated signaling events, such as unfolded protein response (UPR), to the pathophysiology of various neurodegenerative disorders, including Alzheimer’s, Parkinson’s, and Huntington’s disease, amyotrophic lateral sclerosis, and prion disease, is described here. Impaired UPR action is commonly attributed to exacerbated ER stress, pathogenic protein aggregate accumulation, and deteriorating neurodegenerative pathologies. Thus, activating certain UPR components has been shown to alleviate ER stress and its associated neurodegeneration. However, uncontrolled activation of some UPR factors has also been demonstrated to worsen neurodegenerative phenotypes, suggesting that detailed molecular mechanisms around ER stress and its related neurodegenerations should be understood to develop effective therapeutics against aging-associated neurological syndromes. We also discuss current therapeutic endeavors, such as the development of small molecules that selectively target individual UPR components and address ER stress in general.

## 1. Introduction

Most neurodegenerative disorders have common pathological features associated with the abnormal aggregation of misfolded proteins and inclusion bodies in neurons. The toxic buildup of protein aggregates leads to progressive neuronal impairment and loss, culminating in neurodegeneration. Accumulating evidence indicates that alterations of subcellular organelles, particularly the endoplasmic reticulum (ER), are critically involved in pathological neurodegenerative events. In eukaryotic cells, the ER is responsible for around one-third of the total protein synthesis and is highly specialized in the folding and maturation of proteins. The ER also plays a role in protein quality control, a defense mechanism that prevents misfolded proteins from aggregating. However, various environmental challenges can interfere with these ER functions, leading to the accumulation of unfolded or misfolded proteins in the ER lumen, which generates ER stress. In many neurodegenerative diseases, ER stress and the presence of inclusion bodies composed of misfolded protein aggregates are observed early in the symptomatic stage, implying that failure of protein quality control in the ER and disrupted protein homeostasis (proteostasis) contribute to neurodegeneration. Indeed, in animal models, genetic manipulations of ER unfolded protein response (UPR) components have revealed the contribution of the ER stress response in a variety of neurodegenerative disorders. This shows that rectifying ER stress could be a promising therapeutic target for neurodegenerative diseases.

The influence of ER stress on many neurodegenerative diseases, as well as the supporting scientific findings, will be discussed in this review.

## 2. The Neurodegenerative Diseases and Their Common Cellular Events

### 2.1. The Basic Etiologies of the Neurodegenerative Diseases

#### 2.1.1. Alzheimer’s Disease

Alzheimer’s disease (AD) is a gradual and irreversible neurodegenerative disease representing the most prevalent form of dementia. The main clinical features of AD are the progressive deterioration of cognitive functions involving loss of memory and executive function caused by synaptic failure and neuronal loss [1,2]. AD is referred to as a protein misfolding disorder since the prominent neuropathological hallmark of AD is the aggregation and accumulation of misfolded proteins (the formation of amyloid plaques and neurofibrillary tangles) in the brain. The major lesions in AD, leading to synaptic loss and consequential neuronal death, are composed of neurofibrillary tangles (the highly stable polymers of intracellular protein aggregates composed of hyperphosphorylated microtubule-associated tau) and senile plaque (extracellular deposits of insoluble fibrillary amyloid-β (Aβ) peptide, a proteolytic product of amyloid-β precursor protein (APP)) [3,4].

#### 2.1.2. Parkinson’s Disease

Parkinson’s disease (PD) is another prevalent neurodegenerative disease, and more than 90% of patients with PD are sporadic cases. The major clinical features of PD are motor symptoms, including muscle rigidity, tremors, and impaired balance and coordination. Furthermore, dementia is accompanied in many cases. The neuropathophysiological hallmarks of PD are the depletion of striatal dopamine resulting from dopaminergic neuronal loss in the substantia nigra pars compacta (SNpc) and the presence of insoluble cytoplasmic inclusions (called Lew bodies (LB)) that contain misfolded α-synuclein (α-syn) fibrils in the neuron [5,6]. α-syn is a neuronal protein located in the axon terminal of presynaptic neurons and plays a crucial role in synaptic vesicle trafficking and neurotransmitter release [7]. However, if the protein quality control for α-syn is impaired, α-syn assembles to the oligomers and the aggregates, forming insoluble neurotoxic inclusions. The presence of α-syn inclusions is strongly correlated with neuronal damage, and this association is referred to as synucleinopathies. 

Cytotoxic α-syn accumulation leads to many cellular defects, including mitochondrial dysfunction, accumulation of lipid droplets, ROS production, and impaired ubiquitin-proteasomal degradation [8]. Similar to other neurodegenerative diseases, α-syn inclusions give rise to ER stress by altering synaptic vesicle transport, Ca^2+^ homeostasis, intracellular protein trafficking, and ERAD machinery and ultimately result in neurodegeneration [9,10,11]. 

#### 2.1.3. Huntington’s Disease

Huntington’s disease (HD) is a monogenic neurodegenerative disease inherited in an autosomal-dominant manner. HD is clinically characterized by progressive cognitive decline, behavioral and psychiatric disturbances, and motor dysfunction exemplified by involuntary movements throughout the body [12,13]. The pathogenesis of HD proceeds from the accumulation of the large inclusions generated by mutant Huntington protein (huntingtin). The genetic mutation in HD is typically an expansion of CAG trinucleotide repeats in the first exon of the Huntington gene (*HTT*), leading to the translation of abnormally long stretches of aggregation-prone polyglutamine (PolyQ) tract in the N-terminus of the protein. The mutant huntingtins cause neuronal cell death preferentially in the striatum [14,15], a part of the basal ganglia network involved in the execution of cerebral cortex function and motor function. HD disease primarily affects GABAergic neurons in the dorsal striatum, resulting in psychological symptoms such as depression, anxiety, and memory loss [16].

#### 2.1.4. Amyotrophic Lateral Sclerosis

Amyotrophic lateral sclerosis (ALS) (also known as motor neuron disease or Lou Gehrig’s disease) is a neurodegenerative disorder characterized by the progressive degeneration of corticospinal and somatic motor neurons. The selective vulnerability of motor neurons to their denervation results in muscle atrophy, lack of coordination, paralysis of voluntary muscles, and respiratory failure, predisposing to uniform lethality in ALS [17,18]. While the majority of cases (~90%) are classified as adult-onset sporadic ALS (sALS), about 10% of cases are directly inherited familial ALS (fALS) [17,19], affected by more than 50 types of mutation in human genes. The most prevalent genetic mutations are found in superoxide dismutase (*SOD1*), fused in sarcoma (*FUS*), TAR DNA binding protein (*TARDBP*/TDP-43), and chromosome 9 open reading frame (*C9ORF72*) [18,19,20]. 

A common feature of both fALS and sALS is altered proteostasis and the formation of protein inclusions in degenerating motoneurons, among a variety of perturbations of cellular functions in ALS (e.g., altered mRNA metabolism, Ca^2+^ dysregulation, impaired energy production, altered axonal transport, and excessive excitatory tone) [18]. As with other protein folding disorders, ALS-related aberrant protein folding and oligomerization cause ER malfunction and UPR activation, especially in motoneurons [21]. 

#### 2.1.5. Prion Disease

Prion diseases or transmissible spongiform encephalopathies (TSEs) are a category of fatal neurodegenerative disorders caused by the accumulation of structurally abnormal and modified scrapie isoform of prion protein (PrP^Sc^) in the central nervous system [22]. The most remarkable phenomenon in the pathogenesis of prion diseases is the conversion of normal cellular α-helical prion proteins (PrP^C^) into the protease-resistant, misfolded β sheet-rich PrP^Sc^ [23] and the resulting neuronal loss and spongiform degeneration of the brain [24]. Moreover, PrP^Sc^ binds to PrP^C^ and catalyzes its conversion into a cytotoxic modified isoform, accelerating the formation of large PrP^Sc^ aggregates. While this proteinopathy develops throughout the brain, symptoms vary depending on which brain regions are affected by the presence of toxic PrP^Sc^, such as the thalamus in familial insomnia, the cerebral cortex in Creutzfeldt-Jakob disease (CJD), the cerebellum in Gerstmann-Sträussler-Scheinker syndrome (GSS), and the brain stem in dementia and Bovine spongiform encephalopathy (BSE) with psychotic behavior [22]. It has been proposed that the buildup of PrP^Sc^ triggers UPR and causes ER stress-induced cytotoxicity in neurons, leading to prion-associated neurodegeneration similar to other protein misfolding disorders [25].

### 2.2. The Common Cellular Events in the Neurodegenerative Diseases

#### 2.2.1. Calcium Dyshomeostasis

Calcium is critically involved in various intracellular events, functioning as a second messenger; therefore, its concentration is maintained extremely low by actively transporting cytoplasmic calcium out of cells or storing it in the ER or mitochondria. Impaired calcium homeostasis has been implicated in various neurological disorders. In AD, the extracellular Aβ oligomers have been reported to induce extracellular Ca^2+^ influx through the plasma membrane-localized NMDAR and VGCC, contributing to increasing cytoplasmic Ca^2+^ levels and affecting ER Ca^2+^ levels [26,27]. Accordingly, in mature hippocampal neurons, treatment of Aβ oligomers provoked cytosolic Ca^2+^ dyshomeostasis, ER dysfunction, and ER stress-mediated apoptosis [28]. Furthermore, Aβ and presenilin mutations lead to Ca^2+^ dyshomeostasis by inducing ER Ca^2+^ release through ER Ca^2+^ channels, ryanodine receptor (RyR), and inositol 1,4,5-triphosphate receptor (IP_3_R) [29,30,31]. Memantine, an NMDAR blocker, was approved and is utilized to improve cognitive function in people with advanced AD. Through the aforementioned ways, Ca^2+^ dyshomeostasis by elevation of cytoplasmic Ca^2+^ levels under AD eventually results in ER stress and neuronal cell death, further deteriorating AD.

Furthermore, in PD, α-syn oligomers in the plasma membrane form Ca^2+^-permeable pores, allowing Ca^2+^ influx and resulting in PD pathologies and cell death [32]. Additionally, elevated Ca_v_1.3 was observed in dopamine neurons in the SNpc of PD patients, suggesting increased Ca^2+^ influx via Ca_v_1.3 [33]. Accordingly, isradipine, a calcium channel blocker, has been demonstrated to protect dopamine neurons from the toxicity of α-syn oligomers or mitochondrion-targeting neurotoxins such as 1-methyl-4-phenyl-1,2,3,6-tetrahydropyridine (MPTP) [34,35]. Furthermore, PD-associated genes such as *BST1*, *ITPKB*, and *PLA2G6* have been implied to regulate ER Ca^2+^ levels [36,37,38]. Mutation of *LRRK2*, a late-onset familial PD gene, upregulates the expression of mitochondrial Ca^2+^ transporters such as MCU and MICU [39], and accordingly, genetic and pharmacological inhibition of MCU protects dopamine neurons from their loss in mutant PINK1-expressing zebrafish [40].

Mutant proteins causing ALS, HD, and prion diseases also lead to an increase in Ca^2+^ influx and also ER Ca^2+^ dyshomeostasis, which results in excitotoxicity and ER stress in those affected neurons [41,42,43].

#### 2.2.2. Neuroinflammation

Recent findings have documented that neuroinflammation involving glial cells such as astrocytes and microglia critically contributes to neuronal pathologies in neurodegeneration. Microglia are myeloid cells and are involved in the brain’s immune responses. As with peripherally circulating macrophages, microglia exhibit pro-inflammatory (M1 classical activation) or immunoregulatory (M2 alternative activation) responses. M1 microglia express and release pro-inflammatory cytokines such as TNF-α, IL-1β, and IL-6. On the other hand, IL-4 and IL-13 activate M2 microglia, which then release anti-inflammatory cytokines (e.g., IL-10 and TGF-β) [44]. Astrocytes are the most abundant glial cells and are involved in various functions to uphold neuronal integrity and function, including the maintenance of the blood-brain barrier, metabolic support of neurons, and recycling of ions and neurotransmitters [45]. Similar to microglia, astrocytes also display pro-inflammatory or immunoregulatory responses. Pro-inflammatory astrocytes (A1 astrocytes) produce pro-inflammatory factors (e.g., TNF-α, IL-1β, and IL-6), while immunoregulatory astrocytes (A2 astrocytes) make anti-inflammatory factors (e.g., Il-4, IL-10, and TGF-β) [44].

Microglia and astrocytes have been observed to be activated by their contact with neurotoxic aggregates (e.g., Aβ, tau, and α-syn) in vitro, in vivo rodent models, and in the brain samples from human subjects with AD, PD, HD, ALS, or prion disease [44,46,47]. Their activation could be neuroprotective by eliminating protein aggregates [44]. However, the persistent activation of glial cells also results in neurological pathologies. Human genetics studies have suggested that genes involved in the microglial function and other brain immune systems are closely linked to neurodegeneration [48,49]. In addition, microglia and astrocytes have been documented to eliminate synapses via their phagocytic activity [50,51], and activated microglia and astrocytes target neurons to remove synaptic connections and induce neuronal death in vitro and in vivo experimental models of AD [51,52]. Furthermore, elevated pro-inflammatory TNF-α levels are detected in the CSF of patients with AD, although other pro-inflammatory cytokine levels are not so obviously different compared to people without dementia [53,54]. Increased levels of brain cells that are immunoreactive to pro-inflammatory cytokines such as TNF-α, IL-1β, and IL-6 have been found in PD patients [55]. Heightened expression of pro-inflammatory cytokines was also reported in CJD patients’ brains and CSF [47].

Inflammation signaling pathways, including IKK/NF-κB, MAPK (e.g., JNK, p39 MAPK), JAK/STAT, and PI3K/Akt, are involved in producing pro-inflammatory cytokines such as TNF-α and IL-6. On the other hand, IL-1β’s synthesis and release are controlled by the inflammasome. Elevated inflammasome activity such as caspase-1 activity, IL-1β production, and NLRP3 expression has been documented in microglia and neurons in AD, PD, HD, ALS, and prion disease [56]. Furthermore, the activation of NLRP3 inflammasome attenuates Aβ phagocytosis by microglia and promotes Aβ aggregation [57], and also exacerbates tauopathies [58]. NLRP3 deficiency and inhibition of inflammasome activity ameliorate neurodegenerative pathologies in AD and PD [56,57,58].

In sum, neuroinflammation, including glial cell’s action, could be protective against toxic aggregate buildup; however, uncontrolled neuroinflammatory activations may contribute to neurological pathologies.

#### 2.2.3. Autophagy and Mitophagy

Macroautophagy (hereafter referred to as ‘autophagy’) eliminates large cytoplasmic contents, including damaged organelles and protein aggregates. Perturbation of autophagy has been suggested to be associated with aging and age-related neurodegenerative diseases, and genetic studies of autophagy-related genes have demonstrated autophagy’s critical involvement in neurodegenerative diseases [59,60]. Autophagic dysfunction causes the accumulation of the toxic, aggregate-prone proteins that are responsible for neurodegenerative diseases, such as Aβ, tau, a-syn, and mHTT in the brain [61]. Because of their post-mitotic nature, neurons are particularly vulnerable to age-related decline in autophagic capacity and the consequent accumulation of protein aggregates and defective organelles [62]. 

Immature autophagic vacuoles containing a substantial amount of Aβ were observed by electron microscopy in the neurons of AD patients [63,64], and LC3-II colocalized with α-syn-positive-Lewy bodies in PD patients [65,66], while the number of lysosomes and levels and activities of lysosomal enzymes were decreased within dopamine neurons in PD brain [67]. In addition, mHTT expression in HD has shown to induce defects in multiple steps of autophagy like autophagosome biogenesis, autophagic cargo recognition, and retrograde transport of autophagosomes, all of which are crucial for the autophagosome-lysosome fusion along the axon [68,69]. Furthermore, reduced autophagy in skeletal muscle during aging is also known as one of the factors liable to the pathology of motor neuron diseases. An aging-related decline in autophagy-lysosomal activity impairs autophagic flux and exacerbates muscle aging phenotypes and pathologies of ALS [70,71]. Likewise, the age-related autophagic dysfunction and accumulation of disease-associated toxic protein aggregates lead to disruption of the autophagic degradation and contribute to consequent neurotoxicity in the pathology of neurodegenerative diseases [72,73]. 

In PD, impaired mitophagy (autophagy for removing damaged mitochondria) has been implied in the development of neurological pathologies of PD. Human and rodent genetic studies have identified several PD-associated genes [e.g., Parkin (*PRKN*), *PINK1*, DJ-1 (*PARK7*), *GBA*, and *ATP13A2*] and their involvement in autophagy (mitophagy)-lysosome system and PD pathologies [60,74,75,76]. A large number of enlarged, phospho-ERK-labeled mitochondria have been observed within autophagosomes in the SNpc, suggesting compromised mitophagy in PD [77]. Furthermore, the presence of the polyQ tract in mHTT has been reported to impair mitophagy and lead to the accumulation of damaged mitochondria in HD [78].

## 3. ER Protein Quality Control and ER Stress

Under physiological conditions, cytosolic and ER molecular chaperones mediate the precise folding of newly synthesized proteins. In addition, protein quality control mechanisms recognize misfolded proteins, mediate their retention and refolding in the ER, and eventually degrade them through the ER-associated protein degradation (ERAD) pathway if they fail to reach their native structures [79]. These activities minimize the amount of unfolded and misfolded proteins in the ER, preventing aberrant protein aggregation and restoring proteostasis. Fine-tuned protein folding is essential for normal cellular function and survival. However, the fidelity and efficiency of protein folding in the ER are highly influenced by the alterations of intracellular and extracellular stimuli. ER stress can be triggered by a variety of pathological conditions, including overloaded protein synthesis, a disturbed ubiquitin-proteasome pathway, a lack of autophagy, excessive or inadequate nutrients, dysregulated Ca^2+^ or redox homeostasis, inflammatory stimuli, and hypoxia.

In response to ER stress, ER initiates adaptive mechanisms comprising a complex network of signaling pathways, termed the UPR, to re-establish ER homeostasis [80]. Upon the activation of UPR, global translation is initially hampered, thereby decreasing the influx of newly synthesized proteins into the ER. Under moderate accumulation of unfolded proteins, UPR operates as a feedback mechanism, reinforcing protein quality control by increasing the expression of genes, which are generally involved in ER protein folding and the ERAD pathway [81,82,83]. In addition, the ERAD pathway promotes misfolded proteins’ clearance by exporting them to the cytosol, where they are degraded through ubiquitination and proteasomal degradation. UPR signaling also enhances autophagy, which helps to eliminate protein aggregates, especially large ones, through lysosomal degradation.

### 3.1. Key Players in the UPR Pathway

**Figure 1:**
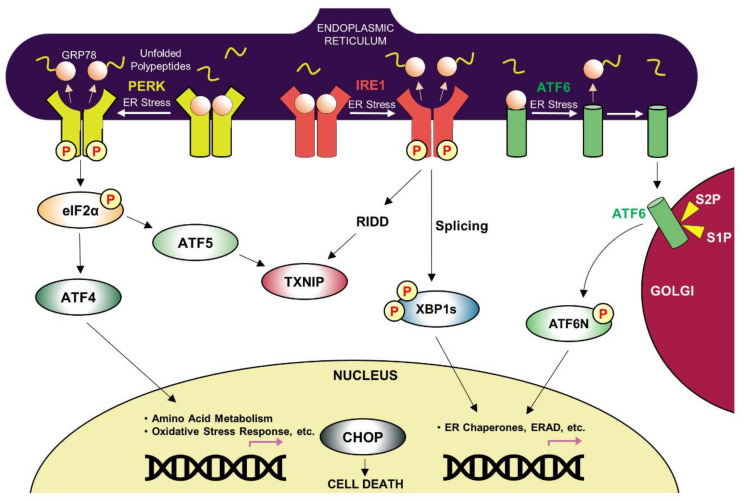
Summary of UPR signaling cascade. UPR is initiated by GRP78’s dissociation from UPR components on the ER membrane under ER stress. PERK and IRE1 undergo autotransphosphorylation after their dimerization or oligomerization, and ATF6 is translocated to Golgi and cleaved by protease S1P and S2P. UPR activation enhances target gene expression to restore ER protein folding capacity or triggers cell death. TXNIP induced by PERK and IRE1 also activates the inflammasome and triggers subsequent cell death.

In response to ER stress, the UPR is initiated by three ER-resident transmembrane proteins: activating transcription factor-6 (ATF6), inositol requiring protein-1 (IRE1), and protein kinase RNA-like ER kinase (PERK) [84,85,86]. These three ER proteins mediate the signaling cascade from the ER lumen to the cytoplasm or nucleus (Figure 1). IRE1 is a type I transmembrane protein that is evolutionarily conserved from the yeast and metazoan cells. IRE1α and IRE1β are two isoforms of mammalian IRE1: IRE1α has been demonstrated to function as a critical UPR factor, while IRE1β, primarily existing in the intestine and lung, has been reported to suppress IRE1α’s activity [87]. Under the unstressed condition, IRE1α exists as a monomer by binding to the ER chaperone GRP78, which suppresses IRE1α activation. However, GRP78 is released from IRE1α in response to the accumulation of unfolded and misfolded proteins in the ER, allowing IRE1α homodimerization or oligomerization. Other studies have also reported that IRE1α homodimerization or oligomerization can be induced by its direct interaction with misfolded proteins [88]. Upon homodimerization or oligomerization, IRE1α autotransphosphorylates itself and activates its RNase domain, which mediates XBP1 mRNA splicing to produce a functional XBP1 protein (spliced XBP1, XBP1s). As a transcription factor, XBP1s travels to the nucleus and enhances the expression of its target genes, the majority of which aid in the restoration of ER homeostasis. Interestingly, XBP1s also functions via protein-protein interaction, not as a transcription factor; previous reports have demonstrated that XBP1s’ physical interaction with FoxO1 suppresses FoxO1′s transcriptional activity [89]. In addition to XBP1 mRNA splicing, IRE1α cleaves and downregulates certain mRNAs and microRNAs with its RNase domain, which is referred to as regulated IRE1-dependent decay (RIDD) [90]. 

Metazoan cells also have PERK, a type I transmembrane protein in the ER. Similar to IRE1α, PERK exists as a monomer by binding to GRP78 under unstressed conditions and undergoes dimerization or oligomerization by unbinding to GRP78 or directly binding to misfolded proteins under ER stress. Subsequent autotransphosphorylation of PERK after its dimerization or oligomerization results in phosphorylation of eIF2α, which attenuates general protein translation to alleviate the burden on the ER. Paradoxically, the translation of specific mRNAs with upstream open reading frames (uORFs) as in *ATF4*, *ATF5*, and CCAAT/enhancer-binding protein α (C/EBPα, *CEBPA*) is elevated. Increased ATF4 levels have been demonstrated to facilitate the expression of growth arrest and DNA-damage-inducible 34 (GADD34, *PPP1R15A*) and C/EBP homologous protein (CHOP, *DDIT3*), both of which have been reported to induce cell death. 

ATF6 is a type II transmembrane protein and a member of the bZIP transcription factor family. Under unstressed conditions, ATF6 is retained in the ER by binding to GRP78. However, ER stress dissociates GRP78 from ATF6, allowing ATF6 to move to the Golgi, where it is cleaved by two proteases, S1P and S2P. Consequently, ATF6′s cytoplasmic region with the bZIP domain translocates to the nucleus and induces its target gene expression, most of which restores ER homeostasis as with XBP1s.

### 3.2. ERAD Pathway and Autophagy-Lysosomal Pathway

**Figure 2:**
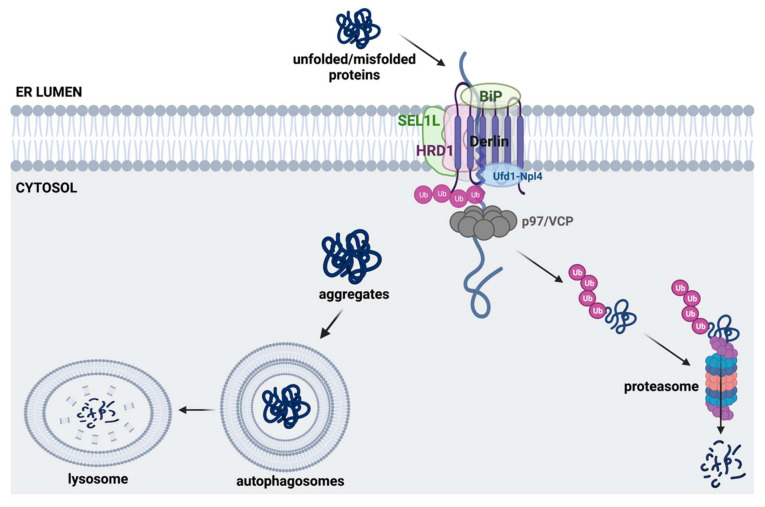
Misfolded protein clearance by autophagy and ERAD. For ERAD, unfolded/misfolded proteins are retrotranslocated to the cytosol through retrotranslocons such as Derlin and Hrd1, and ubiquitinated by E3 ligase (e.g., Hrd1; Sel1 is a cofactor of Hrd1). Polyubiquitinated ERAD substrate proteins are recognized and degraded by 26S proteasome in the cytosol. Protein aggregates, macromolecules, and organelles are also cleared via the autophagy-lysosomal pathway.

The UPR’s initial attempt is to restore ER homeostasis by increasing the protein folding capacity in the ER. However, misfolded ER proteins that do not attain their native structures are ultimately disposed of by ERAD and autophagy [91]. Because numerous neurodegenerative diseases are caused by impaired proteostasis, combined responses of UPR, ERAD, and autophagy are critical to maintaining ER homeostasis and preventing neurodegenerative pathologies. Under ERAD, misfolded ER proteins need to be translocated out of the ER due to a lack of a ubiquitin-proteasome system (UPS) in the ER lumen (Figure 2). The misfolded ER proteins are recognized and linearized by ER chaperones and retrotranslocated from the ER lumen through retrotranslocons such as Sec61, Derlins, gp78, and Hrd1 with the help of an ATPase, p97/VCP. Retrotranslocated misfolded ER proteins are subsequently ubiquitinated by ER-resident E3 ligases such as Hrd1 and gp78 and ultimately degraded by 26S proteasomes [92]. 

Autophagy is another way to get rid of intracellular materials, particularly macromolecules and organelles. Initially, autophagy was identified as a survival mechanism by consuming internal materials in the absence of external nutrients, but it was also revealed later to be involved in numerous cellular events, notably including the turnover of damaged or aged intracellular organelles [93]. Autophagy proceeds via sophisticated autophagic machinery centrally controlled by the mechanistic target of rapamycin (mTOR) and AMP-activated protein kinase (AMPK). mTOR (mTORC1 in mammalian cells) suppresses autophagy by inhibiting ATG1 (Unc-51 like autophagy activating kinase 1 (ULK1) in mammals) activity, while AMPK promotes autophagy by downregulating mTORC1 activity and also directly activating ULK1. ER stress and its associated signaling events such as UPR have been documented to be critically involved in autophagy [94]. ER stress-induced IRE1α activation leads to autophagy via its interaction with tumor necrosis factor receptor-associated factor 2 (TRAF2) and subsequent activation of c-Jun N-terminal kinases (JNKs) [95]. Additionally, the PERK-ATF4-CHOP pathway has been demonstrated to elevate autophagic gene expression [96]. Recent studies have also documented that ER stress induces autophagic turnover of the ER (ER-Phagy) as an ER quality control [97]. Protein clearance processes such as the UPS and autophagy decline with aging, contributing to aging-associated disorders such as neurodegenerative diseases [98,99].

### 3.3. ER stress, Inflammation, and Cell Death

When the aforementioned ER quality control systems (UPR, ERAD, autophagy) fail to restore ER homeostasis, the UPR triggers cell death to prevent the damaging effect of accumulated misfolded proteins on neighboring cells. While PERK’s initial response is to attenuate protein translation, PERK-mediated induction of ATF4 and CHOP leads to apoptosis by elevating protein synthesis [100]. Furthermore, IRE1α’s activity is controlled by its physical interaction with pro-apoptotic BAX and BAK, but IRE1α also triggers inflammation and cell death by its association with TRAF2 and ASK1 [101,102]. Conversely, p38 MAPK and IKKβ phosphorylate XBP1s, which leads to XBP1s nuclear translocation and its activation [84,103,104]. In addition, PERK and IRE1α lead to cell death by inducing *TXNIP* expression and subsequent inflammasome activation (Figure 1) [105]. PERK’s downstream transcription factor ATF5 increases *TXNIP*’s expression, while IRE1α’s RIDD activity downregulates miRNA (miR-17), reversing miR-17’s inhibitory action on *TXNIP*’s translation [84]. ER stress leads to astrocyte’s inflammatory responses by activating JAK1/STAT3 signaling and increasing pro-inflammatory cytokine expression such as IL-6, CCL2, and CCL20, which is dependent on the PERK pathway [106]. 

### 3.4. ER Dysfunction and Neurodegenerative Diseases

**Figure 3:**
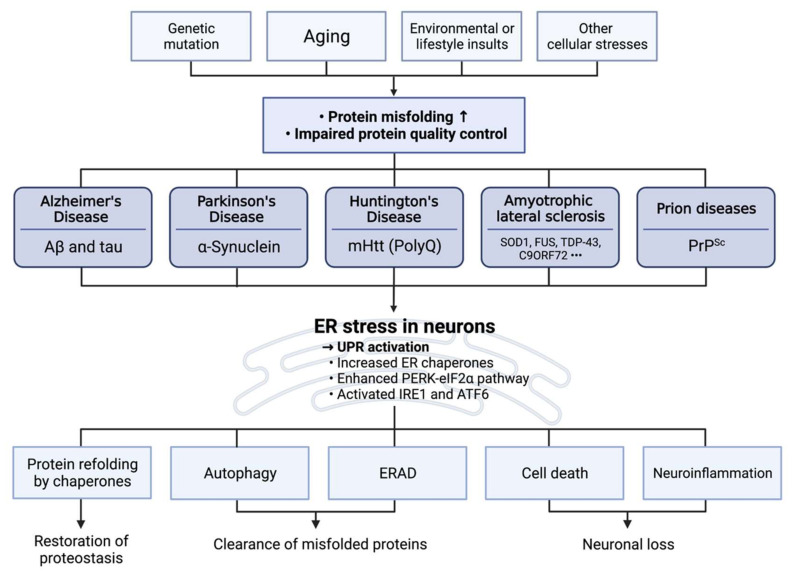
ER stress and neurodegeneration triggered by misfolded protein aggregates. Genetic mutations, aging, environmental insults, and various cellular stresses disrupt ER protein quality control and proper folding of proteins. Increased protein misfolding induces ER stress and accelerates the accumulation of disease-associated protein aggregates. ER chaperone activities and UPR pathways are enhanced as adaptive stress responses to alleviate ER stress. As a result, various chaperones and protein clearance mechanisms such as autophagy and ERAD contribute to refolding or eliminating misfolded proteins. However, when sustained ER stress in the neuron exceeds the capacity of adaptive responses to cope with protein misfolding, ER stress can lead to neuronal cell death and neuroinflammation, contributing to the development of neurodegenerative pathologies.

Neurons, the post-mitotic cell, are particularly susceptible to the toxic effects of mutated or misfolded protein accumulation, requiring appropriate protein quality control and stress responses such as UPR upon various environmental perturbations (Figure 3). As mentioned above, neurodegenerative diseases are marked by the accumulation of misfolded protein aggregates in the neuron. Although this pathophysiology is reasonably attributed to the mutation of specific proteins that escape protein quality control mechanisms, it is noteworthy that age is the critical risk factor for most neurodegenerative disorders. Deterioration of proteasomal degradation and the increased reactive oxygen species during aging could contribute to the decline in clearance and the enhanced production of misfolded proteins [107,108]. On this account, the cell death induced by UPR and the ER stress substantially contributes to the pathological development of several age-associated neurodegenerative diseases (Figure 3).

We will document the contribution of ER dysfunction and ER stress response to the etiology of neurodegenerative diseases such as Alzheimer’s disease, Parkinson’s disease, Huntington’s disease, amyotrophic lateral sclerosis, and prion disease.

## 4. Alzheimer’s Disease

### 4.1. ER UPR on Neuronal Pathophysiology in AD

Numerous studies have reported the elevation of ER UPR in the AD brain. The PERK-eIF2α pathway is hyperactive in the brain of animal AD models and postmortem brain samples from patients with AD [109,110,111,112]. Furthermore, increased GRP78/BiP expression was detected in AD patients’ temporal cortex and hippocampus, the fundamental regions responsible for cognition and memory [113]. Furthermore, elevated IRE1α phosphorylation was also observed in the hippocampal neurons of patients with AD and colocalized with abnormally phosphorylated tau [114,115]. Increased ATF4 expression was also detected in axons in the AD brain, and mechanistically ATF4 was proposed to act as a mediator for spreading Aβ pathology [116]. In addition, elevated phosphorylation of IRE1α and PERK was observed in neurons and glial cells in people’s brains with tauopathies [117].

However, there are contradictory perspectives regarding the balance between the protective and destructive role of the UPR in AD pathology. In the early stages of AD pathology, activated UPR could operate as a defensive response to rescue neurons by expanding the folding capacity of the ER with increasing molecular chaperones and enhancing the degradation of protein aggregates by ERAD and autophagy [118,119]. An in vitro study has demonstrated that Aβ treatment in neuronal cells induced the PERK-eIF2α pathway, and silencing PERK expression enhanced neuronal cell death while enhancing eIF2α phosphorylation by salubrinal (eIF2α dephosphorylation inhibitor) alleviated it [120]. Additionally, several reports have implied that XBP1s may play a cytoprotective role against toxic aggregates in a variety of Aβ-associated AD models, including Aβ-expressing *Drosophila* and Aβ-treated cultured mammalian neurons [121], and also in tauopathy-related AD models, including transgenic *Drosophila* and *C. elegans* expressing aggregation-prone mutant tau variants [122,123]. Furthermore, in the Chinese Han population, the -116C/G polymorphism of XBP1 has been linked to AD susceptibility [124]. 

On the other hand, excessive ER stress and prolonged UPR activation can be detrimental to the neurons as UPR could contribute to worsening neurodegeneration via the apoptotic pathway activation. IRE1α has been discovered to interact with Presenilin1 (PS1), a protein known to cleave APP to Aβ, which leads to the activation of the JNK/c-Jun pathway, implying a link between amyloid accumulation and neuronal death in AD [125]. Moreover, the enhanced JNK3 was found in the brain and cerebrospinal fluid of patients with AD. It was correlated with Aβ levels, implicating its contribution to the aggravation of AD pathologies, including cognitive decline [126].

### 4.2. The Age-Associated Decline of ER Capacity and AD

The single most important risk factor for AD is aging. In addition, the age-associated disruption in neuronal physiology also frequently accompanies ER dysfunction and aberrant proteostasis that might result from excessive accumulation of protein aggregates. The decline in the UPR and cellular clearance capacity with advancing age is marked by downregulation of chaperone activity, increased reactive oxygen species [108], and diminished ERAD pathway [107], all of which lead to disempowering of ER’s ability to maintain proteostasis [127,128,129,130]. For example, reduced ER molecular chaperone levels such as GRP78/BiP, calnexin, and PDI have been observed in the aged hippocampus and other brain regions such as the cortex and cerebellum [131]. Furthermore, the aging brain displays reduced *PERK* mRNA transcription and eIF2α phosphorylation [131,132,133]. Reduced UPR responses in the aged brain are often accompanied by elevated proapoptotic responses involving CHOP/GADD153 and caspase-12 [131]. Paradoxically, activated UPR, such as elevated phosphorylation of PERK and eIF2α, are often reported in the affected brain areas of patients with AD [115]. It is still unclear if observed UPR activation in AD indicates the UPR’s pathogenic functions or the compensatory response to elevated ER stress.

In addition, AD-related protein aggregates such as Aβ and tau also elicit the inhibition of proteasome activity, further contributing to proteostasis disruption and neuronal degeneration [134,135]. This might explain the observed reduction in proteasome activity in the brains of patients with AD [134]. Furthermore, selective dysfunction of proteasomal degradation toward AD-causing proteins is documented: phosphorylated tau could be ubiquitinated and degraded by interacting with Hsp90 and CHIP, a chaperone-E3 ligase complex [136]. However, FK506 binding protein 51 kDa (FKBP51) could block Hsp90-CHIP-mediated tau degradation, and its expression elevates with advancing age and is correlated with AD progression [137]. 

Unlike early-onset AD, sporadic AD barely has identified genetic mutations involved in the Aβ production and accumulation. Thus, the foremost cause triggering Aβ accumulation in sporadic AD might be an imbalance between Aβ production and degradation. Autophagic vacuoles are not typical in the healthy brain, but autophagic vacuoles containing a substantial amount of Aβ are abundantly observed in the neurons in the early stage of AD even before Aβ is extracellularly deposited [63,64]. This implies that the decline in autophagosome-lysosome function during age may contribute to Aβ deposits and AD pathologies [64]. For example, Beclin 1, one of the key players in autophagy, is lowered in the brain of patients with early-stage AD, and the reduction in Beclin 1 expression is also correlated with age [138,139]. In mice, the heterozygous deletion of Beclin 1 leads to reduced neuronal autophagy, aberrant lysosomal structure, Aβ accumulation, and neurodegenerative pathologies in the cortex and hippocampal areas [139].

### 4.3. UPR Components and Their Role in Memory, Cognition, and Synaptic Plasticity in AD

Active synthesis of new proteins and their translational control are critical for long-lasting synaptic plasticity, long-term memory consolidation, and additional neuronal functions, including neuronal growth and axonal guidance [140,141]. The UPR could act as a negative regulator of synaptic plasticity through the phosphorylation of eIF2α and reduction in protein translation [142]. However, the UPR animal models exhibited complicated neuronal phenotypes: forebrain-specific *Perk*-deficient mice demonstrated deficits in fear extinction memory and cognitive function while suppressing ATF4 action in the forebrain resulted in enhanced long-term synaptic plasticity and memory in mice [143,144]. Furthermore, *Gcn2*-deficient mice displayed enhanced spatial memory of weak conditioning but also showed its deficit after more intense training [141]. 

Dysregulated neuronal protein translation could also contribute to AD-related cognitive impairments. Indeed, attenuation of PERK, GCN2, and PKR signaling ameliorates β-amyloidosis, neurodegeneration, and other AD-related deficits in synaptic plasticity, spatial memory, and cognition in AD model mice [145,146,147,148,149]. In a recent study, ISRIB, a newly developed small molecule that binds to and stabilizes eIF2B, reversed the effect of phosphorylated eIF2α, restored hippocampal protein synthesis, and rescued impaired long-term memory and synaptic function in the mouse model of AD [150]. 

Other components of the UPR have also been documented to be involved in AD pathophysiology. IRE1α deficiency in the nervous system leads to Aβ plaque deposit reduction and attenuated astrocyte activation in the cortex and hippocampus and improves cognitive capacity in AD model mice [151]. Paradoxically, neural-specific *Xbp1* deletion impairs the process related to contextual memory formation and long-term potentiation, whereas forced neural expression of XBP1s improves the aforementioned processes and synaptic transmission in the hippocampus [152]. Moreover, the hippocampal expression of XBP1s in an AD mouse model via a lentiviral vector also leads to the recovery of long-term memory formation, dendritic spine density, and hippocampal synaptic transmission [153]. Although *Xbp1* deficiency in the hippocampus does not alter the expression of typical UPR component genes, XBP1s modulates the expression of genes related to memory formation, dendritic function, and synaptic activity, including GABAergic markers and brain-derived growth factor (*Bdnf*) [152,154]. This implies that XBP1s plays a direct role in a molecular network in cognitive processes under physiological conditions and AD.

## 5. Parkinson’s Disease

### 5.1. ER Stress and α-Synuclein-Related PD Pathology

The α-synuclein-related ER stress has been documented in various in vitro and in vivo models of PD; the yeast synucleinopathy model [11], the PD patient-derived induced pluripotent stem cells (iPSCs) [155,156], a mouse model of α-syn toxicity (A53TαSTg) [157], and the postmortem brain tissue from patients with PD [158]. The upregulation of ER chaperones, including BiP, PDI, and homocysteine-induced ER protein (Herp), and their colocalization with LB were observed in the SNpc of human PD brain tissue [157,159,160]. In addition, increased phosphorylation of PERK and eIF2α were detected in cultured cells treated with PD-inducing neurotoxin [161,162] and in dopaminergic neurons of the SNpc from patients with PD [163]. Phosphorylated PERK is also colocalized with α-syn inclusions in dopaminergic neurons [163,164], suggesting the close association of UPR activation with the α-syn aggregation. Accordingly, forced expression of human α-syn in rat SNpc induced UPR activations, including increased levels of ATF4, nuclear ATF6, CHOP, XBP1s, and phosphorylated eIF2α [165]. Conversely, GRP78/BiP expression in rat SNpc alleviated α-syn-induced neurotoxicity, increasing dopaminergic neurons’ survival and striatal dopamine levels [165]. Additionally, in in vitro and in vivo models of neurotoxin-induced parkinsonism, in which neurotoxins such as 6-hydroxy-dopamine (6-OHDA) and MPTP are typically administered, the genetic ablation of *Ddit3* (CHOP) [166] and overexpression of XBP1s [167,168] protect neurons from neurotoxin-induced cell death. 

The pathological form of α-syn could be released from the affected neurons and found in the extracellular compartments and body fluids, including plasma [169], the cerebrospinal fluid, urine, saliva, and tears [169,170,171,172,173,174,175,176]. The transmission of α-syn to neighboring neurons promotes intracellular α-syn aggregation and the formation of LB inclusions, leading to recipient cells’ death and further progressing PD [177,178]. Protein folding stress could facilitate α-syn release and transmission to neighboring neurons. For example, proteasome and lysosome inhibitors trigger the α-syn release from cells via non-canonical exocytosis [179]. 

Disruption in protein clearance mechanisms is also a significant contributor to ER stress and the pathophysiology of PD. Under physiological conditions, α-syn can be degraded by the chaperone-mediated autophagic pathway, macroautophagy, and UPS [180]. However, in PD, accumulation of α-syn impairs autophagy [181,182] as well as proteasomal degradation [183]. Human patients with PD exhibit reduced nuclear levels of transcription factor EB (TFEB), a major regulator of the autophagy-lysosome pathway, in dopaminergic neurons in SNpc [184]. Likewise, forced α-syn expression disrupts the nuclear localization of TFEB and autophagy [184]. In addition, the α-syn undergoes various posttranslational modifications, disrupting the autophagic degradation of α-syn and other substrates [185]. Conversely, stimulation of autophagy with forced expression of TFEB or Beclin 1 restores the clearance of inclusions and elicits protection against α-syn-induced neurotoxicity [184]. 

### 5.2. Genetic Mutations in PD Related to ER Stress

Autosomal recessive juvenile parkinsonism (AR-JP) is strongly linked to the genetic mutations in Parkin (*PRKN*) [186], an E3 ubiquitin ligase involved in the regulation of mitophagy. Parkin has been proposed to protect cells from ER stress-induced cell death [187]. Overexpressing Parkin protects dopaminergic SH-SY5Y cells from ER-stress-induced mitochondrial dysfunction and cell death [187,188], whereas downregulation of Parkin makes cells highly vulnerable to ER stress [188]. Mechanistically, Parkin ubiquitinates the insoluble forms of GPR37 (also called Parkin-associated endothelin-like receptor (Pael-R)) and promotes their degradation, thus preventing GPR37-mediated cell death induced by unfolded protein stresses [189]. Additionally, Parkin could protect cells from ER stress by upregulating XBP1 expression through transcriptional repression of p53 that has suppressed XBP1 expression [190]. In addition to Parkin’s protective role under ER stress, mitochondrial and ER stress increase Parkin transcription via ATF4’s binding to the Parkin promoter [188]. 

### 5.3. Mitochondrial Dysfunction and Calcium Dyshomeostasis in ER Stress-Related PD Pathophysiology

Mitochondria interact physically and functionally with the ER. This inter-organelle interaction mediates various physiological processes and the viability of cells by primarily modulating Ca^2+^ signaling and the execution of the cell death pathway [191]. ER stress can induce mitochondrial damage and vice versa, and such an interconnection between two organelles has been implicated in PD pathogenesis. Mitochondrial toxins, such as rotenone, MPTP, or its active derivative MPP+, and 6-OHDA also cause ER stress and UPR activation [161,162,192]. As previously mentioned, ER and mitochondrial stress up-regulate Parkin expression through ATF4, and Parkin protects cells from mitochondrial or ER stress-induced cell death [188]. 

In healthy neurons, an increase in intracellular Ca^2+^ triggers the neurotransmitter release from the presynaptic terminal. Besides the influx of Ca^2+^ through voltage-dependent Ca^2+^ channels in the plasma membrane, the ER is another primary source of Ca^2+^ in the cell [193]. The fluctuations of Ca^2+^ levels exceeding or below the physiological range in the cytoplasm can be detrimental to the survival of dopamine neurons [34,194]. However, in pathological conditions with continual cellular stress, neuronal homeostasis could be threatened by Ca^2+^ dyshomeostasis [193]. MPP+ induced ER stress and triggered Ca^2+^ release from the ER and concomitant Ca^2+^ uptake into the mitochondria. Elevated Ca^2+^ in the mitochondrial matrix impaired mitochondrial membrane potential, causing caspase activation and consequent cell death [195]. Likewise, inhibiting ER Ca^2+^ release with RyR antagonist dantrolene prevented MPP+-mediated caspase activation [195].

Non-pathological α-syn mediates Ca^2+^ transfer from the ER to the mitochondria by associating with mitochondria-associated ER membranes (MAM) and promoting the ER-mitochondria interaction [196,197]. Conversely, pathological α-syn loses its association with MAM, leading to a decline in MAM function and mitochondrial Ca^2+^ uptake, mitochondrial fragmentation, and a subsequent increase in mitophagy [196,197].

## 6. Huntington’s Disease

### 6.1. Mutant Huntington Protein in the Pathogenesis of HD

Like other neurodegenerative diseases, HD has a feature of neurotoxic protein inclusions containing the mutant protein aggregates. Huntingtins are subject to proteolytic cleavage, generating more toxic and aggregation-prone fragments [198,199]. The majority of huntingtins are found in the cytoplasm [200]; however, N- and C-terminal huntingtin fragments are abundant in the nucleus [201]. The intranuclear insoluble huntingtin inclusions were more pronounced in neurons of HD patient brain [202], cultured cells, and transgenic mice expressing mutant human huntingtin (mHTT) [203,204]. N-terminal huntingtin fragments derived from normal polyQ repeats interact with the nuclear pore protein translocated promoter region (TPR) for their shuttling between the nucleus and cytoplasm. However, N-terminal fragments with expanded polyQ are weakly bound to TPR, leading to poor nuclear export and accumulation in the nucleus [205]. This mutant fragment’s intranuclear aggregation induces nuclear abnormalities, neuronal cytotoxicity, and eventual neurodegeneration in HD [206,207,208]. Mechanistically, mutant huntingtins manifest abnormal transcriptional repression and altered expression of multiple genes [201,209,210]. PolyQ motif in transcription factors mediates the interaction with other transcriptional regulators, and forming the aggregates with expanded polyQ of those transcription factors has been demonstrated to interfere with proper gene transcription [209]. The intranuclear huntingtin aggregates hinder transcriptional regulation through the sequestration of transcription factors, such as TATA-binding protein, p53, and CREB-binding protein, into huntingtin-containing inclusions [211,212,213]. Furthermore, marked reductions in mRNAs encoding neurotransmitter receptors, such as glutamate and dopamine receptors, were observed in the pathogenic human mHTT-expressing HD mouse models [214,215]. These findings suggest that the pathogenic mutant huntingtins interfere with normal neuronal functions and cause HD by disrupting nuclear organization and transcriptional regulation.

### 6.2. Impact of Pathogenic Mutant Huntingtins on ER Stress

Studies using *C. elegans* and *Drosophila* expressing proteostasis sensors have revealed that the expression of expanded polyQ disrupts cellular proteostasis [216,217]. Furthermore, several in vitro and in vivo studies have documented that heat shock proteins such as Hsp40 and Hsp70 families prevent insoluble huntingtin aggregate formation [218,219], protect neurons from toxic aggregate-induced cell death [220], and alleviate neurodegenerative phenotypes [221,222,223]. Conversely, Hsp40 and Hsp70 expression is reduced in human mHTT expressing mouse brains [224]. 

In addition, the pathogenic huntingtin oligomers with expanded polyQ interfere with UPS and ERAD, resulting in ER stress [225]. Although huntingtin is not a canonical ER-localized protein, Hrd1 and gp78, E3 ligases involved in ERAD, have been demonstrated to ubiquitinate mutant huntingtins and promote their proteasomal degradation with the help of p97/VCP that disintegrates polyQ-containing huntingtin aggregates [226,227,228]. However, when UPS and ERAD are overwhelmed by the increased formation of mutant huntingtin aggregates, huntingtin aggregates sequester p97/VCP [229,230] and interfere with the functions of gp78 and p97/VCP [228]. Furthermore, other ERAD proteins, Npl4 and Ufd1, are also sequestered by mutant huntingtin fragments, thus contributing to polyQ toxicity. At the same time, Npl4 and Ufd1 expression ameliorate polyQ toxicity in vitro studies using yeast and PC12 cells [229]. 

The striatal cells from another HD mouse model (Hdh^Q111^ knock-in mouse), in which *Hdh* (mouse homolog of human *HTT*) was modified to have expanded CAG repeats, exhibit elevated p53 levels and an enlarged ER [231,232]. Accordingly, increased ER stress/UPR markers were observed in cultured cells harboring pathogenic huntingtins (human mHTT, Hdh^Q111^), striata of Hdh^Q111^ mouse, and parietal cortex of human HD postmortem brains [229,232,233,234]. 

Even though elevated UPR marker levels in HD suggest increased ER stress, whether UPR activation protects neurons from HD-related pathologies depends on the different roles of UPR factors. In HD mouse models and HD patient samples, ATF6 processing (its cleavage at the Golgi for activation) is impaired, which may predispose neurons to ER stress [235]. Moreover, striatal cell lines expressing Hdh^Q111^ and striatal neurons of Hdh^Q111^ mouse exhibit increased eIF2α phosphorylation [233]. This increased eIF2α phosphorylation in HD likely suggests elevated ER stress rather than the PERK-eIF2α pathway’s pathological contribution. Indeed, pharmacological activation of the PERK pathway indirectly by salubrinal or directly by various PERK activators (A4, CCT020312, MK-28) protects pathogenic huntingtin-expressing cells from ER stress-induced cell death [233,234,236]. Interestingly, mHTT-expressing mice with neuronal *Xbp1* deficiency display alleviated HD pathologies, including improved neuronal survival and motor performance. This improvement from *Xbp1* deficiency is mechanistically attributed to enhanced expression of FoxO1 and elevated macroautophagy [237]. Additionally, pathogenic huntingtins were shown to trigger ER stress-induced cell death via IRE1-TRAF2-ASK1 complex and JNK activation [101].

## 7. Amyotrophic Lateral Sclerosis

### Proteostasis Disturbances and ER Stress in ALS Pathology

The hypertrophied cell body and proximal axon hillock were observed in the spinal motor neurons of ALS mouse models in early studies [238]. These mice also had dense clumps of neurofilaments and ubiquitin immunoreactive inclusions in swollen axons of the spinal cord, comparable to those described in human ALS, as well as disrupted proteostasis [239,240,241]. iPSCs-derived motoneurons from patients with ALS carrying mutations of *SOD1* also develop common pathological features as well as increased oxidative stress, mitochondrial dysfunction, and elevated ER stress/UPR [242]. Furthermore, irregular morphology of the ER, including dilatation, fragmentation, and distension of rough ER with ribosome detachment, were observed in the spinal anterior horn cells from postmortem tissues [243]. Thus, ER stress is one of the earliest pathological signatures driving the degeneration of motoneurons in ALS [244,245,246]. Indeed, impaired proteostasis and elevated ER stress are frequently observed in the postmortem tissues of fALS and sALS patients, as well as in the cellular and animal models of the disease [247,248,249,250]. In transgenic mice expressing the SOD1 mutation, intraluminal retention of high molecular weight aggregates of SOD1 protein were found in the spinal cord motor neuron ER and colocalized with ER chaperones. The accumulated SOD1 aggregates in the ER may trigger ER stress and other inclusion-induced pathological processes [248,251,252]. Accordingly, the activation of all branches of the UPR pathway in motor neurons is also a noticeable feature in ALS pathology in vitro and in vivo. The level of IRE1α phosphorylation and subsequent *Xbp1* mRNA splicing were higher in the spinal cord motoneurons of symptomatic SOD1-G93A mice [248] and SOD1-G85R mutant-expressing cells [253]. The activation of the PERK pathway (PERK and eIF2α phosphorylation; ATF4 and CHOP expression) was also observed in various ALS models, including mutant SOD1-expressing mice [245,246,254], ALS-related mutants (SOD1, FUS, TDP-43)-expressing cells [253,255,256], *Drosophila* model expressing TDP-43 aggregates [257], and patients’ spinal cord samples with sALS [247]. Elevated CHOP expression was detected both in neurons and glial cells of the spinal cords from patients with sporadic ALS and mice expressing mutant SOD1 [258]. In addition, the cleavage and nuclear translocation of ATF6 were enhanced in vitro and in vivo ALS models [248,253]. Besides, mutant SOD1 physically interacts with Derlin-1, leading to disturbance of retrotranslocation of ERAD substrates from the ER to the cytosol, thereby interrupting ERAD-mediated clearance of ER luminal misfolded proteins and triggering ER stress in motor neurons [259]. This mutant SOD1 also induces an IRE1-TRAF2-ASK1 pathway-dependent apoptotic pathway, contributing to the neurodegeneration under ALS [259].

## 8. Prion Disease

### ER Stress and UPS Impairment in Prion Diseases

ER chaperon expression and ER stress-induced caspase-12 activation were significantly increased in neuronal cell lines treated with purified PrP^Sc^ from scrapie-infected mice brains or postmortem brain samples of patients with CJD [260]. Transcriptional analysis in BSE also documented the upregulated expression of cytosolic chaperones (Hsp70 and DnaJ), as well as ER chaperones (GRP94, GRP170, and GRP78/BiP) [261]. Many other genes in UPS and autophagy-lysosome pathway were also increased in brain samples from BSE-infected animals, demonstrating that pathogenic prion proteins evoked ER stress [261]. In addition, an augmented PDI expression was observed in prion-infected mice and the brain of patients with sporadic CJD [262]. Likewise, in pathogenic prion-expressing cells and mouse brains, as well as in the brain tissues of patients with CJD, an increase in ERp57, another PDI family protein (also called GRP58), was detected [263,264]. Interestingly, ERp57 expression ameliorated PrP^Sc^-induced toxicity, while ERp57 silencing exacerbated prion-associated pathologies in PrP^Sc^-expressing cells, and these were proposed to be mediated via the physical interaction between ERp57 and PrP^Sc^ [263,264].

Additional ER stress could exacerbate the cytotoxicity and neurodegeneration caused by PrP^Sc^. Treatment of proteasome inhibitors and ER stress-inducing agents leads to the extensive accumulation of insoluble PrP^Sc^ aggregates [265,266]. In contrast, as in ERp57, UPR activations, including IRE1α, XBP1, ATF6, and ATF4, attenuate PrP aggregate formation [266]. The persistent activation of the PERK-eIF2α pathway and subsequent repression of protein translation are induced by pathogenic prions’ accumulation. On the other hand, restoring protein translation by dephosphorylating eIF2α with GADD34 or inhibiting PERK by a pharmacological agent (GSK2606414) alleviates synaptic deficits and neuronal loss in the prion-infected mouse brain, whereas salubrinal administration worsens prion-induced neurotoxicity [267,268].

As in pathogenic Aβ, tau, and huntingtin, disease-associated PrP^Sc^ also impairs UPS. Mechanistically, PrP^Sc^ specifically inhibits the catalytic β subunit of the 26S proteasome and its proteolytic activity [269], which would further disrupt proteostasis and contribute to prion-associated neurodegeneration.

## 9. Future Perspectives—Therapeutic Strategies for Targeting ER Stress and Neurodegenerative Diseases

Protein misfolding and toxic buildup of aggregates are the pathological hallmarks of various neurodegenerative diseases. Thus, targeting protein quality control mechanisms, such as protein folding, ER stress responses, and clearance of misfolded proteins, might be a plausible therapeutic strategy.

### 9.1. Targeting Protein Misfolding and ER Stress

Preventing toxic inclusion formation by directly targeting misfolded protein aggregates or molecular chaperons could be a promising option to treat protein misfolding-associated neurodegenerative disorders. Numerous chemicals are identified to function as “chemical chaperones” and alleviate ER stress (Table 1). For example, 4-phenylbutyric acid (4-PBA) and tauroursodeoxycholic acid (TUDCA) have been suggested to act as chemical chaperones by binding to exposed hydrophobic residues of unfolded proteins. However, 4-PBA and TUDCA may also attenuate ER stress via other unknown mechanisms.

Other small molecules have also been identified to resolve or prevent Aβ and tau aggregation, likely by acting as chemical chaperones: Congo red, polyphenol-based compounds, curcumin, and thioflavin-T for targeting Aβ aggregates [270]; methylene blue, curcumin derivatives, N744, rhodanines, and aminothienopyridazines for tau aggregates [271]. Likewise, Congo red, trehalose, polyphenol-based compounds, and C2-8 have been demonstrated to inhibit mHTT aggregate formation [272], while polyphenol-based compounds, curcumin, myricetin, tanshinones, and ginsenoside Rb1 have been shown to suppress α-synuclein oligomerization [273]. Using structural information of prions and in silico drug screening, several small molecules such as a diphenylmethane derivative (GN8), a carbazole derivative (5Y), and small aromatic molecules (NPRs) have been identified to bind to prions and prevent them from forming aggregates [274]. Methylene blue also showed neuroprotective and ER stress-suppressing properties in ALS model organisms such as *C.elegans* and zebrafish expressing human mutant FUS or TDP-43 [275,276]. Remarkably, a recent clinical trial has successfully demonstrated that 4-PBA and TUDCA effectively reduce cell death and slow the functional decline of motor neurons in patients with ALS [277].

### 9.2. Targeting UPR Components

Recent efforts have identified several small molecules specifically targeting UPR factors such as PERK, eIF2α, GADD34, IRE1α, and ATF6 [278] (Table 2). In particular, the PERK branch has emerged as an effective target for several neurodegenerative diseases, including AD, PD, ALS, and prion disease [234,279,280,281]. Salubrinal has been reported to exert a protective role against ER stress-induced neuronal death in PD and HD [234,282]. However, salubrinal might be inappropriate for long-term treatment since it could impair spatial long-term memory formation due to sustained repression of protein translation [283]. Selective PERK activators, MK-28 and CCT020312, have been shown to ameliorate neuronal toxicity from mHTT and tau [236,284]. Additionally, phenotyping screening has identified SB1617, which activates PERK and ameliorates tauopathies [268]. In contrast, the oral administration of PERK inhibitor GSK2606414, restored global protein translation and slowed prion disease progression with neuroprotection throughout the brain [268]. Likewise, recently identified ISRIB restores protein translation and provides neuroprotection in prion-infected and pathogenic Aβ-expressing mice [150,285] and also in mutant SOD1-expressing primary neurons [286]. Another PERK inhibitor, SC79 induces Akt-mediated PERK phosphorylation at Thr799, preventing eIF2α phosphorylation and displaying neuroprotection in prion-infected mice [287].

Another branch of UPR, the IRE1-XBP1s pathway, has also been proposed as a promising target to address ER stress-associated diseases. A recent study has identified several lead compounds that specifically activate IRE1-XBP1s signaling through high-throughput screening and transcriptional profiling [294]. These selected compounds inhibit mutant APP secretion and promote APP-degradation via ERAD. Another study discovered small molecules that inhibit PDI and attenuate mHTT- and Aβ-induced neurotoxicity [296].

### 9.3. Future Perspectives

Increased life expectancy due to medical advancements, along with declining birth rates, has resulted in the globe becoming an aging civilization. Chronic metabolic disorders and neurological diseases such as AD, PD, HD, and ALS become more common as a result of this. The majority of neurodegenerative illnesses are caused by the accumulation of misfolded protein aggregates, which are accompanied by a disruption of proteostasis (Figure 3). However, there has not been a treatment for these neurodegenerative disorders directly addressing impaired proteostasis. Recent studies have documented that ER homeostasis and its impairment (ER stress) are crucially involved in aging-associated diseases such as metabolic and neurodegenerative diseases. The mechanistic relevance of ER stress and its related signaling events (UPR) in proteostasis and neurodegenerative disorders has been studied and explored here. Furthermore, we have introduced recent progress on the development of ER stress-targeting therapeutics. ER stress-relieving compounds such as 4-PBA and TUDCA have shown their therapeutic promises not only in various neurodegenerative animal models but also in recent clinical trials. Developing specific UPR-targeting molecules would be promising, but they also face potential problems such as conflicting outcomes and concerns over their long-term safety. As an alternative approach, phenotypic drug screening could be helpful to find new small molecules to target ER stress and its associated pathologies. For example, a connective map (CMAP) provides in silico screening of small molecules using gene expression changes as a phenotypic assay, which has been beneficial for finding novel drugs such as celastrol and withaferin A to target cellular stress-induced pathologies [85]. Indeed, celastrol, which was initially identified with CMAP to ameliorate ER stress and its associated diseases such as obesity [297], has also been demonstrated to alleviate ER stress-related neurodegenerative diseases [298]. Additionally, the recent development of targeted protein degradation methods such as lysosome-targeting chimera (LYTAC) and proteolysis-targeting chimera (PROTAC) would make it possible to target previously undruggable UPR factors, protein aggregates, and other ER stress-related proteins [299].

## Figures and Tables

**Table 1:** Chemical chaperones and other small molecules targeting pathogenic protein aggregations.

Disease	Affected Brain Regions	Disease-Causing Protein Deposited/Mutant	Effective Chemical Chaperones and Other Small Molecules
Alzheimer disease	Cortex, hippocampus, basal forebrain, brain stem	Amyloid β peptide derived from APP/ mutation in APP, presenilin1 or presenilin2, APOE4 allele	Congo red, polyphenol-based compounds, curcumin, thioflavin-T
		Hyperphosphorylated tau	Curcumin derivatives (e.g.,Dibenzoylmethane), methylene blue, N744, rhodanines, aminothienopyridazines (ATPZs)
Parkinson disease	Substantia nigra, cortex, locus coeruleus, raphe, etc.	α-Synuclein	Polyphenol-based compounds, curcumin, myricetin, tanshinones, ginsenoside Rb1
Huntington disease	Cortex, striatum, other basal ganglia, etc.	Huntington with polyglutamine expansion (exon1)	Congo red, trehalose, C2-8
Amyotrophic lateral sclerosis	Spinal motor neurons and motor cortex	Mutations in C9orf72 (40~50%), SOD1 (20~25%), TDP-43 (4~5%), FUS (4~5%), etc.	4-PBA, TUDCA, methylene blue
Prion disease	Cortex, thalamus, brain stem, cerebellum, etc.	Prion protein (PrP^Sc^)	Diphenylmethane derivative (GN8), carbazole derivative (5y), small aromatic molecules (NPRs)

**Table 2:** UPR component targeting molecules and their reported efficacy on protein aggregates-related neurological pathology.

UPR Target	Molecule		Target Pathology	Reference
PERK signaling activators	*CCT020312*	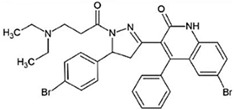	HD, tauopathy	[236,284]
	*MK-28*	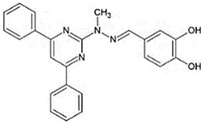		
eIF2α phosphatase inhibitors	*Salubrinal*	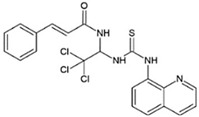	HD, α-synucleinopathies	[157,234,282]
	*Sephin1*	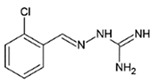	ALS	[288]
	*Guanabenz*	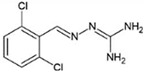	ALS	[289,290]
PERK kinase inhibitor	*GSK2606414*	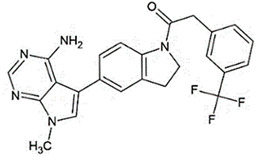	tau-related pathology (AD, frontotemporal dementia), Prion, PD, Marinesco-Sjögren syndrome, ALS	[257,268,279,280,281,291]
Downstream inhibitors of PERK signaling	*ISRIB*	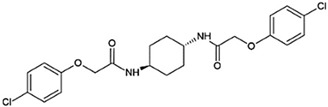	AD, Prion	[150,292]
	*Trazodone*	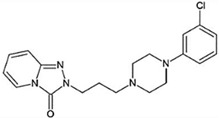	Prion, tauopathy-frontotemporal dementia	[293]
	*Dibenzoylmethane*	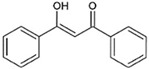		
IRE1/XBP1s activation	*IXA1*	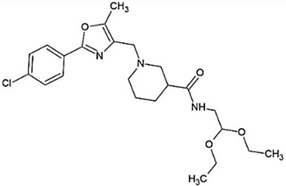	AD	[294]
	*IXA4*	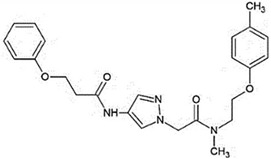		
	*IXA6*	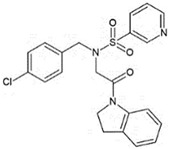		
Activation of ATF6 transcriptional activity	*AA147* *(N-(2-Hydroxy-5-methylphenyl)-3-phenylpropanamide)*	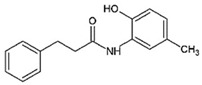	Amyloid aggregates-related pathology	[295]

## Abbreviations

4-PBA	4-phenylbutyric acid
6-OHDA	6-hydroxy-dopamine
AD	Alzheimer’s disease
ALS	Amyotrophic lateral sclerosis
AMPK	AMP-activated protein kinase
APP	Amyloid-β precursor protein
AR-JP	Autosomal recessive juvenile parkinsonism
ASK1	Apoptotic-signaling kinase-1
ATF4	Activating transcription factor-4
ATG	AuTophaGy(ATG)-related proteins
ATP13A2	ATPase Cation Transporting 13A2
Aβ	Amyloid-β
BAK	Bcl-2 homologous antagonist killer
BAX	Bcl-2-associated X protein
BDNF	Brain-derived growth factor
BSE	Bovine spongiform encephalopathy
BST1	Bone Marrow Stromal Cell Antigen 1
C/EBPa	CCAAT/enhancer binding protein a
C9ORF72	Chromosome 9 open reading frame
CHIP	Carboxy-terminus of Hsc70 interacting protein
CHOP	C/EBP homologous protein
CJD	Creutzfeldt-Jakob disease
CREB	cAMP-response element binding protein
eIF2a	Eukaryotic translation initiation factor α
ER	Endoplasmic reticulum
ERAD	ER-associated protein degradation
ERK	Extracellular signal-regulated kinase
ERp57	ER protein 57
FKBP51	FK506 binding protein 51 kDa
FoxO1	Forkhead box protein O1
FUS	Fused in sarcoma
GABA	γ-Aminobutyric acid
GADD153	Growth arrest and DNA-damage-inducible 153
GBA	Glucosylceramidase Beta
GCN2	General control nonderepressible 2
Gp78	Glycoprotein 78
GRP78/BiP	78 KDa glucose-regulated protein/
HD	Huntington’s disease
Herp	Homocysteine-induced ER protein
Hrd1	HMG-CoA reductase degradation protein 1
Hsp90	Heat shock proteins 90
HTT/mHtt	Huntington/mutant Huntington
IKK/NFκB	IkappaB kinase/Nuclear factor kappa B
IL-	Interleukin-
IP_3_R	Inositol 1,4,5-triphosphate receptor
IRE1	Inositol requiring protein-1
ISRIB	Integrated stress response inhibitor
ITPKB	Inositol-Trisphosphate 3-Kinase B
JAK/STAT	Janus kinase/Signal transducer and activator of transcription
JNK/c-Jun	c-Jun N-terminal kinase/c-Jun
LB	Lew bodies
LRRK2	Leucine-rich repeat kinase 2
MAM	Mitochondria-associated ER membrane
MAPK	Mitogen-activated protein kinase
MCU	Mitochondrial calcium uniporter
MICU	Mitochondrial Calcium Uptake
MPP+	1-methyl-4-phenylpyridinium
MPTP	N-methyl-4-phenyl-1,2,3,6-tetrahydropyridine
mTOR	Mechanistic target of rapamycin
NLRP3	NLR Family Pyrin Domain Containing 3
NMDAR	N-methyl-d-aspartate receptor
Npl4	Nuclear protein localization protein 4
Pael-R	Parkin-associated endothelin-like receptor
PD	Parkinson’s disease
PDI	Protein disulfide isomerase
PERK	Protein kinase RNA-like ER kinase
PI3K	Phosphoinositide 3-kinase
PINK1	PTEN-induced kinase 1
PKR	Protein kinase R
PLA2G6	Phospholipase A2 Group VI
PolyQ	Polyglutamine
PrP^C^/PrP^Sc^	Cellular α-helical prion proteins/Scrapie isoform of prion protein
PS1	Presenilin1
RIDD	Regulated IRE1-dependent decay
RyR	Ryanodine receptor
S1P/S2P	Site 1 protease/Site 2 protease
SNpc	Substantia nigra pars compacta
SOD1	Superoxide dismutase
TARDBP/TDP-43	TAR DNA binding protein
TFEB	Transcription factor EB
TNF-	Tumor necrosis factor-
TPR	Translocated promoter region
TRAF2	Tumor necrosis factor receptor-associated factor 2
TSEs	Transmissible spongiform encephalopathies
TUDCA	Tauroursodeoxycholic acid
TXNIP	Thioredoxin interacting protein
Ufd1	Ubiquitin recognition factor in ER associated degradation 1
ULK1	Unc-51 like autophagy activating kinase 1
uORFs	Upstream open reading frames
UPR	Unfolded protein response
UPS	Ubiquitin-proteasome system
VCP	Valosin-containing protein
VGCC	Voltage-gated calcium channel
XBP1	X-box binding protein 1
α-syn	α-synuclein
